# A patient costing analysis of Lesotho’s cervical cancer screening program

**DOI:** 10.3389/fpubh.2026.1888325

**Published:** 2026-10-14

**Authors:** Thatohatsi Sefuthi, Cari Van Schalkwyk, Lungiswa Nkonki

**Affiliations:** 1 Division of Health Systems and Public Health, Global Health Department, Stellenbosch University, Stellenbosch, South Africa; 2 South African Centre for Epidemiological Modeling and Analysis (SACEMA), Centre for Epidemic Response and Innovation (CERI), School for Data Science and Computational Thinking, Stellenbosch University, Stellenbosch, South Africa; 3 Department of Public Health, Walter Sizulu University, Mthatha, South Africa

**Keywords:** cervical cancer screening, health economics, low-and-middle-income-countries, patient costs, screen-and-treat

## Abstract

**Background:**

Cervical cancer is a leading cause of cancer-related mortality worldwide and disproportionately affects low- and middle-income countries. While cervical cancer elimination strategies increasingly emphasize primary HPV testing and screen-and-treat approaches, limited evidence exists on the costs incurred by patients accessing these services. Patient-incurred costs may pose barriers to accessing screen-and-treat services, particularly in resource-constrained settings such as Lesotho.

**Materials and methods:**

This study estimated patient costs for cervical cancer screening and treatment at the Senkatana Cervical Cancer Center of Excellence in Lesotho. A survey was conducted among 100 women accessing cervical cancer screening services between January and November 2025. Data was collected on direct, indirect, and productivity costs incurred by patients and accompanying caregivers. Screening modalities included Visual Inspection with Acetic Acid (VIA), HPV DNA testing, and cytology (Pap smear testing), with eligible patients receiving Loop Electrosurgical Excision Procedure (LEEP) treatment. Costs were converted to international US dollars using 2024 purchasing power parity estimates. A one-way sensitivity analysis was conducted to identify patient cost drivers.

**Results:**

Among the 100 women interviewed, 46% were screened with Pap smear testing, 44% with HPV DNA testing, 9% with VIA, and 3% received LEEP treatment. The majority (91%) were required to return to the facility to collect results, with a mean turnaround time of 32.4 days. The mean total cost per patient per visit was $21.4 (95% CI: $ 17.42–$ 25.98.). Indirect costs accounted for the largest share at $11.05 (52%), followed by productivity costs at $9.40 (44%). Direct medical costs contributed $0.95 (4%). Extrapolated costs across the screening-to-treatment cascade were $62.3 for Pap-LEEP, $61.7 for HPV-LEEP, and $41.2 for VIA-LEEP, representing approximately 17%, 17%, and 11% of average monthly income, respectively. A one-way sensitivity analysis identified indirect medical costs as the primary cost driver, followed by productivity costs.

**Conclusion:**

Patient-incurred costs associated with cervical cancer screening and pre-cancer treatment are substantial. Indirect costs and productivity losses represent critical demand-side barriers that are insufficiently captured in provider-focused evaluations. Incorporating patient costs into cervical cancer screening policy, program design, and economic evaluations is essential to ensure that elimination strategies are clinically effective, accessible, and equitable.

## 1 Introduction

Cervical cancer is the fourth-leading cause of cancer mortality among women worldwide, with 662,301 new cases and 348,874 deaths reported in 2022 (1). The disease disproportionately affects LMICs, with 85% of women with cervical cancer living in poorer countries (2). In sub-Saharan Africa, western, middle, and southern African countries are most affected by cervical cancer mortality, with world-age standardized mortality rates of 16, 22.9, and 29.4 per 100,000 women-years, respectively (3). In Lesotho, a landlocked country by South Africa, cervical cancer is the most common female malignancy, resulting in 413 deaths in 2022 (1). With an HIV prevalence of 19.3% among adults and a disproportionately higher prevalence of 24.3% among Basotho women aged 15–49 years, Basotho women are at a markedly higher risk of cervical cancer incidence and faster progression; making cervical cancer prevention a priority in Lesotho (4, 5).

In 2020, the World Health Organization (WHO) published the “Global Strategy to Accelerate the Elimination of Cervical Cancer as a Public Health Problem”, which targets elimination (cervical cancer incidence below 4 per 100,000 women-years) by 2,120 (2). To achieve elimination, the strategy sets 2030 targets: a) full vaccination with the nonvalent HPV vaccine in 90% of girls by age 15; b) 70% of women screened at ages 35 and 45 using an optimal test; and c) 90% of those with pre-cancerous lesions treated and invasive cancers managed (2). Achieving these targets requires not only the availability of effective screening technologies but also the design of service delivery models that facilitate equitable access, continuity of care, and completion of the screening-to-treatment cascade (6). In alignment with this, Lesotho launched an organized screening program at the Senkatana Cervical Cancer Center of Excellence in February 2013, with support from the United States Agency for International Development (USAID) and the Elizabeth Glaser Pediatric AIDS Foundation (EGPAF) (7). The center provides Papanicolaou smear (Pap smear) testing, HPV DNA testing, and Visual Inspection with Acetic Acid (VIA). For eligible patients, HPV DNA testing serves as the primary screening modality. Introduced in Lesotho in 2025, HPV DNA testing has since achieved facility coverage of 23.1% (8).

While expanding screening and treatment services is an important step toward cervical cancer elimination, these services incur costs for both the health system and patients. Understanding the full economic implications of cervical cancer service delivery is critical to optimizing program design, ensuring sustainability, and maximizing population-level impact (9). This understanding is particularly important in a country such as Lesotho, where healthcare has historically been financed through a blend of domestic financing and significant external aid, which is rapidly shrinking (10). To this end, several studies have examined cervical cancer screening and treatment costs, predominantly focusing on provider costs, including capital investments, consumables, personnel, and laboratory expenses (11–13). Table 1 summarizes the findings on provider costs from studies conducted in Senegal, Taiwan, and Burkina Faso.

**Table 1:** Summary of screening and treatment provider costs.

Study	Country	Findings
Diop et al. (11)	Senegal	VIA cost/screen: $3.71, Pap smear cost/screen: $16.49, HPV DNA testing cost/screen: $46.65. Thermoablation cost/treatment: $32.35, Cryotherapy cost/treatment: $47.35, LEEP cost/treatment: $195.24.
Tang et al. (12)	Taiwan	CIN1 management costs: NT$4,201, CIN3 management costs: NT$14,406, Stage 1 ICC management costs: NT$210,230 Stage 4 ICC management costs: NT$464,701
Devine et al. (13)	Burkina Faso	VIA/VILI cost/screen: $3.5 and careHPV cost/screen: $23

Evidence from multiple low- and middle-income (LMIC) settings indicates that visual inspection with acetic acid (VIA) screening is the least costly, whereas HPV DNA screening incurs the highest costs (9). However, VIA is less cost-effective than HPV DNA due to its inferior diagnostic properties (14). While this literature provides insight into screening costs and cost drivers associated with deploying various screening strategies, it presents an incomplete picture of the economic burden of cervical cancer screening and the treatment of precancerous lesions, because it excludes patient costs. Patient costs, including out-of-pocket payments, transportation expenses, time costs, and productivity losses, influence health-seeking behavior and screening uptake (15). These demand-side costs may be pronounced for screening services that require multiple visits to health facilities.

Despite their importance, patient costs associated with cervical cancer screening and pre-cancer treatment remain understudied in LMIC settings. A limited body of evidence in LMICs measures these costs. Vodicka et al. (9) report the costs of integrating pre-cancer care and cervical cancer treatment in an HIV clinic in Kenya. Sriram et al. (15) report patient costs associated with HPV screening in India, without comparing them with those of other screening modalities. A few additional studies in Iran, Brazil, and Eswatini provide societal estimates of the cost of illness (16–18). However, these analyses do not capture the full screening-to-precancer-treatment cascade and focus on advanced disease rather than prevention.

Limited patient cost data represents a critical gap in the evidence base for cervical cancer screening policy and program design. Without a clear understanding of the economic burden on women, screening strategies may be optimized for technical efficiency but be misaligned with patient affordability and on-ground access. This gap is particularly consequential in high-unemployment settings, where opportunity costs and lost income may substantially deter preventive health-seeking behavior.

This study sought to address country-level knowledge gaps in the costs of cervical cancer screening and treatment services, using the Senkatana Cervical Cancer Screening Center as the study setting. The authors adopted a patient perspective and analyzed screening and treatment costs incurred by patients. The reported findings are descriptive, and no statistical comparisons were made. To the authors’ knowledge, this is the first study in Lesotho to assess patient costs associated with cervical cancer screening and pre-cancer treatment.

## 2 Materials and methods

### 2.1 Study setting

Lesotho delivers health services across primary, secondary, and tertiary levels. The country has 286 health facilities, of which the government owns 121 (10). The Senkatana Cervical Cancer Center of Excellence, a high-volume facility in Maseru, Lesotho, provides walk-in and referral services for cervical cancer screening, pre-cancer treatment, and the diagnosis of invasive cancers (19). Patients are screened using VIA, HPV DNA testing, or cytology through Pap smear testing. The screening modality selection is contingent on the women’s health profiles (20). Eligible patients with precancerous lesions are treated with the Loop Electrosurgical Excision Procedure (LEEP) (20). Patients requiring oncology services are referred to hospitals in South Africa (Universitas and Pelenomi) and India at the Lesotho government’s expense, with approximately 7,000,000 USD spent on patient treatment costs (10, 21). As a government-owned institution, the center subsidizes cervical cancer screening and treatment of precancerous lesions, and patients do not pay user fees for these services. However, patients incur costs for transportation, time, and lost income when accessing care.

During registration, patients are profiled using clinical variables such as age and number of births to inform screening modality selection. After registration and screening selection, patients are referred to the appropriate clinical examination rooms, where nurses collect screening test samples. For VIA testing, nurses collect the sample, apply acetic acid, immediately interpret the results, and then share the results with the patient regarding the presence or absence of abnormal cells. In contrast, for Cytology (Pap smear testing) and HPV DNA testing, nurses collect the samples and send them to the National Reference Laboratory (NRL) for processing. Patients are expected to return to the center to collect their results once they are available.

### 2.2 Sample size estimation and data collection

The patient sample size was estimated to measure patient transport costs with precision. Using the formula (*Zσ*/*e*)^2^, where *Z* is the critical value from the standard normal distribution corresponding to the chosen confidence level, *σ* is the standard deviation of the outcome variable, and *e* is the desired margin of error. The standard deviation of patient transport costs was derived from preliminary, unpublished data based on public transport fares from the Lesotho Transport Association. At 95% confidence and a 5% margin of error, the calculation yielded a sample size of 100 women.

From January to November 2025, patient cost data were collected through a structured, interviewer-administered survey administered to 100 first-time visitors to the cervical cancer unit for screening services. A consecutive sampling approach was used, in which, all eligible first-time attendees were invited to participate until the target sample size of 100 women was reached. The survey captured direct medical costs, direct non-medical costs, indirect costs, and productivity losses incurred by patients and accompanying caregivers (Appendix 1). Eligible participants were identified by clinic staff and referred for interview participation. All participants provided both verbal and written informed consent before data collection.

### 2.3 Costing approach

All reported costs were originally collected in Lesotho Maloti, and converted to international dollars (US) using Lesotho’s 2024 purchasing power parity (PPP) conversion factor of 5.90 (22) allowing for like-for-like comparisons of the relative cost of resources across different country settings (23). No inflation adjustment was applied because all costs were collected within a single study year, and costs measured within the same time period are directly comparable without further adjustment (23). Using the human capital approach, productivity losses were based on travel time and waiting time, and valued using Lesotho’s minimum hourly wage of LSL 14.38 (24). For the 43% of unemployed participants, the minimum hourly wage was applied as a replacement cost proxy for opportunity cost, thereby avoiding the assignment of zero value to their time (25). For participants in industry or manufacturing, earnings are tied to hourly attendance. Subsequently, their self-reported lost income was captured. A bootstrap analysis was performed using non-parametric sampling with replacement (10,000 iterations) on sampling-weighted cost data (26). Ninety-five percent confidence intervals were estimated using the percentile method, based on the 2.5th and 97.5th percentiles of the bootstrap distribution. The generated cost per patient per visit was used to extrapolate costs across the screen-to-treat cascade of care.

### 2.4 Sensitivity analysis

To assess the robustness of the base-case cost estimates and identify key cost drivers, a one-way (univariate) sensitivity analysis was conducted (27). Separate one-way (univariate) sensitivity analyses were performed for the mean cost per patient and the extrapolated screen-and-treat cascade costs. A one-way sensitivity analysis was selected because it provides a foundation for understanding the structure of the cost analysis before considering more complex multivariate approaches (27). The resulting lower and upper estimates were calculated by decreasing and increasing each base-case cost component by 20%, respectively, and recalculating the total cost outcome while all other parameters remained unchanged. Given the absence of published confidence intervals for all cost parameters in this setting, a uniform ±20% range was applied, representing plausible extremes, and consistent with standard practice for assessing parameter-level influence (28, 29). Correlations among parameters are not accounted for in this form of analysis, an acknowledged limitation of the univariate approach (29). However, the present analysis measures distinct cost categories without drawing any correlations.

### 2.5 Classification and valuation of patient costs

Patient-incurred costs were classified into direct medical costs, direct non-medical costs, and productivity costs (9). Direct medical costs included any out-of-pocket expenditures incurred during the screening visit, such as payments for medications or diagnostic services not covered by the facility. Direct non-medical costs captured expenses related to accessing care, including transport, food, and accommodation where applicable. Productivity costs reflected the loss of economic output due to time patients and accompanying caregivers lost during clinic visits, including travel time, waiting time, and time spent receiving services.

Productivity losses were estimated using self-reported travel and waiting times, along with associated income loss. For participants engaged in paid employment, lost income was calculated based on reported wages. For participants engaged in informal employment or unpaid work, productivity losses were valued using self-reported estimates of forgone income or economic activity.

### 2.6 Ethical approval

Before data collection, ethical approval for the study was obtained from the Stellenbosch University Health Research Ethics Committee (ID S24/04/096 (PhD)) and the Lesotho Ministry of Health Research Coordinating Unit (ID 294-2024 RENEW 01).

## 3 Results

### 3.1 Socioeconomic demographics

Table 2 summarizes the respondents’ socioeconomic characteristics. The sample of interviewed women had a mean age of 45.69 (range: 19–94 years), with a modal age of 38 years. Half of the respondents were married, and 43% were unemployed. Among employed women, nearly one-third (31.6%) worked in the informal or manual sector, followed by commerce and administration (19.3%), and industrial or manufacturing occupations (15.8%). Educational attainment was generally low, with only 29% of women having completed tertiary education. The average family size was 4.76 (range: 1–12).

**Table 2:** Socioeconomic demographics.

Variable	Category	Frequency (*n* = 100)	Percentage (%)
Age in years	15–24	3	3%
	25–54	74	74%
	55–64	11	11%
	65+	12	12%
Marital status	Single	21	21%
	Married	50	50%
	Divorced	8	8%
	Widowed	19	19%
	Others	2	2%
Employment status	Unemployed	43	43%
	Part-time employed	1	1%
	Full-time employed	34	34%
	Self-employed	22	22%
Occupation	Agriculture	2	2%
	Building and construction	2	2%
	Health and social care	3	3%
	Education and training	8	8%
	Industrial/Manufacturing	9	9%
	Informal/Manual	18	18%
	Commerce and administration	11	11%
	Food and hospitality	4	4%
Education level	None	1	1%
	Primary	39	39%
	Secondary	31	31%
	Tertiary	29	29%
Family size	0–5	79	79%
	6–10	17	17%
	11+	4	4%

### 3.2 Service delivery characteristics

Among the participants, 97% (*n* = 97) received cervical cancer screening services, while 3% (*n* = 3) received LEEP treatment, necessitated by a diagnosis of precancerous lesions through prior screening. Most women underwent laboratory-based screening with Pap smear (46.4%) and HPV DNA testing (44.3%), accounting for over 90% of all screening tests, whereas only 9.3% underwent VIA screening. Women who had been screened for cervical cancer through HPV DNA testing and Pap smear testing, constituting 90.7% (*n* = 88) of the sample size reported being required to return to the care setting to receive screening results. Women who were screened through VIA (9.3%; *n* = 9) received same-day results. Based on the provisional return dates provided by facility staff, the mean turnaround time (TAT) for screening results was estimated at 32.4 days.

Figure 1 illustrates screening TAT by screening modality. VIA had the fastest results turnaround time, with patients receiving a same-day diagnosis. The mean TAT for HPV DNA testing was 33.8 days, with a narrow interquartile range of 28 to 29 days, though there were outliers. Pap smear testing had a mean TAT of 37.5 days; the TAT variability ranged from 2 to 181 days.

**Figure 1:**
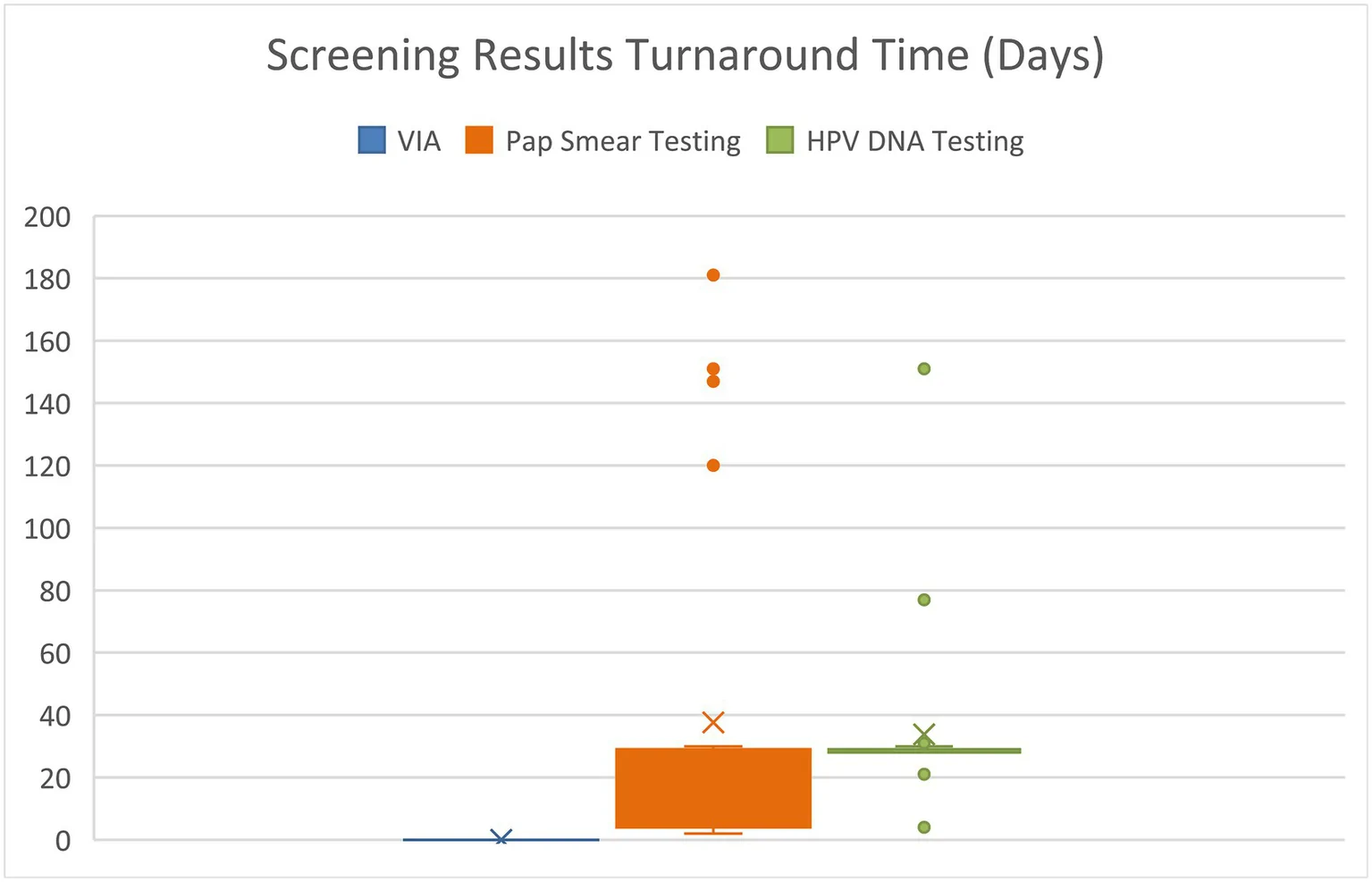
Screening results turnaround time (days).

Overall, the average TAT for screening results was 32.4 days (range: 0–181 days) with a modal waiting time of 28 days.

### 3.3 Patient costs

Most participants (61%) utilized public transportation to reach the cervical cancer center, 24% used private means, and 15% walked. The average travel time was 1.3 h, with a range from 0.17 to 48 h. Regarding costs, 80% reported expenses are associated with reaching the center, while 20% incurred none. The majority (80%) went to the center alone, while 20% were accompanied by a caregiver. Approximately 35% of the participants reported losing income due to attending care, while 65% reported not having lost income. Table 3 summarizes patient-incurred costs per visit.

**Table 3:** Patient costs per visit.

Cost component	Mean cost ($)	Mean cost bootstrap ($)	Standard error	95% (CI)
Direct medical costs				
Patient out of pocket	0.31	0.31	0.03	0.26–0.38
Pap smear testing	0.64	0.64	0.26	0.51–1.49
Sub-total	0.95	0.96	0.25	0.51–1.49
Indirect costs				
Patient transport	10.30	10.27	1.66	7.34–13.83
Non-medical expenses when undergoing treatment (e.g., meals)	0.75	0.75	0.23	0.34–1.25
Sub-total	11.05	11.03	1.76	7.68–15.08
Productivity costs				
Travel time costs (patients)	2.30	2.30	0.20	1.94–2.72
Waiting time costs (patients)	2.62	2.62	0.22	2.21–3.06
Time spent by caregivers	1.33	1.33	0.31	0.76–1.97
Reported income loss	3.15	3.13	1.13	1.13–5.57
Sub-total	9.4	9.39	1.37	7.06–12.41
Mean total cost/patient	21.4	21.37	2.20	17.42–25.98

Data revealed that, while services are free, some women opted to pay for Pap smear sample processing at private laboratories to achieve a shorter results turnaround time, which is reflected in the total cost. The mean direct medical cost per patient was $0.95 (95% CI: $ 0.51–1.49) and contributed 4% of the total cost incurred by patients. The mean indirect cost was $11.05 (95% CI: 7.68–15.08) and contributed 52% of the total cost. The mean productivity cost was $9.4 (95% CI: 7.06–12.41) and accounted for 44% of the total mean cost/patient.

### 3.4 Estimated costs per screen and treat strategy

Using on single-visit cost data, we extrapolated patient costs for three screening and treatment approaches, assuming that productivity and indirect costs were tripled for HPV- and Pap-based testing and doubled for VIA-based testing. This assumption is grounded in observed clinical pathway differences associated with each screening modality. Women undergoing VIA screening receive their results the same day and, if eligible, are expected to return for a follow-up visit to receive treatment. Women undergoing Pap smear or HPV DNA testing have screening samples collected and sent for laboratory processing. Women receive screening results at a second visit, once available, and treatment appointments are scheduled only after these results are received, necessitating an additional visit for treatment. As a result, women incur varied indirect and productivity costs, depending on the type of screening they undergo. Figure 2 summarizes the extrapolated costs. Additionally, a one-way sensitivity analysis (Table 4) was conducted to assess the variation in the visit multiplier. Based on the observed patient costs per visit along with the assumed number of visits, the extrapolated completed pathways costs were $62.3 for Pap-LEEP, $61.7 for HPV-LEEP, and $41.2 for VIA-LEEP.

**Figure 2:**
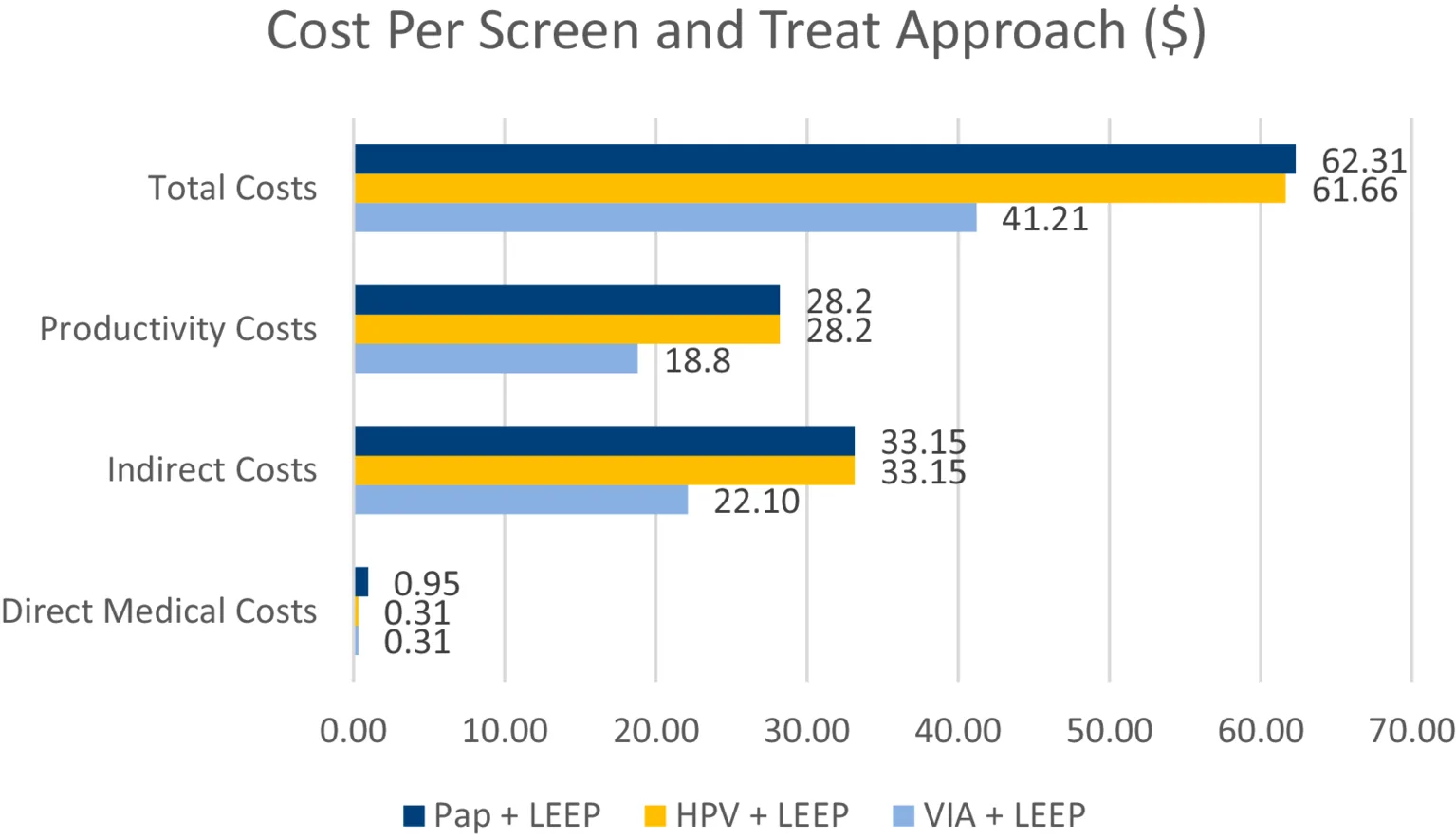
Extrapolated costs per woman per screen-and-treat pathway.

**Table 4:** Patient costs per visit one-way sensitivity analysis.

Cost component	Base case ($)	Low (−20%) ($)	High (+20%) ($)	Absolute change
Direct medical costs	0.95	0.76	1.14	0.38
Indirect costs	11.05	8.84	13.26	4.42
Productivity costs	9.4	7.52	11.28	3.76

### 3.5 Sensitivity analysis results

Table 4 and Figure 3 show the results of the one-way sensitivity analysis for patient costs per visit. The tornado diagram illustrates Table 4, showing the range of total costs generated by varying each cost component by ±20% around its base-case value while holding all other parameters constant. Indirect medical costs had the greatest sensitivity, with an absolute change of 4.42, closely followed by productivity costs at 3.76. Direct costs had the smallest sensitivity (absolute change: 0.38).

**Figure 3:**
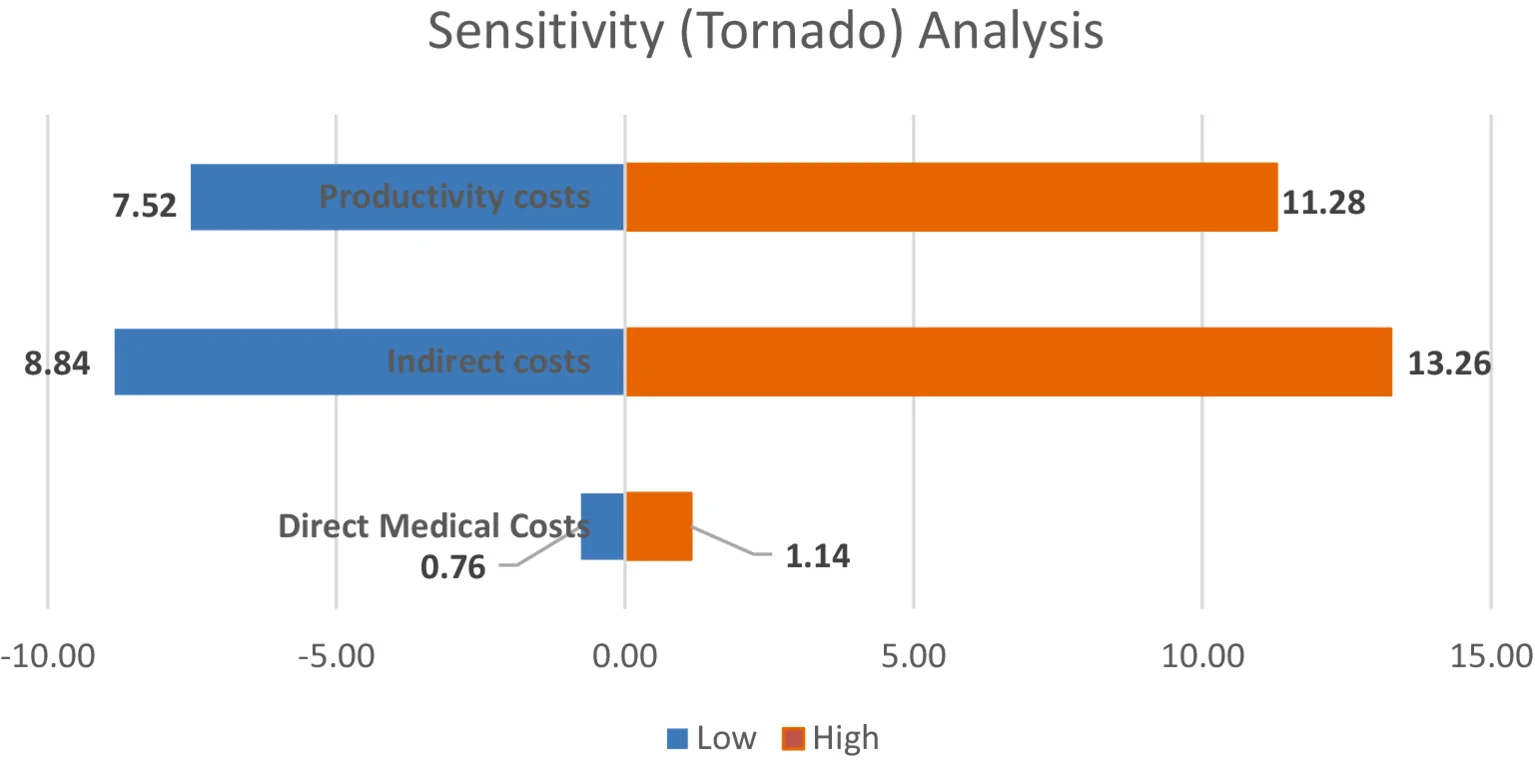
Patient costs per visit tornado diagram.

## 4 Discussion

This study estimated the costs incurred by patients for cervical precancer screening and treatment services at the Senkatana Cervical Cancer Center of Excellence. The findings demonstrate that patients incur indirect costs and productivity losses, despite the absence of user fees. Furthermore, findings show that differences in clinical pathways across the screening modalities contribute to variations in patient costs, with VIA-based screening strategies leading to lower indirect and productivity costs due to the provision of same-day screening results. In contrast, Pap smear and HPV DNA- based testing approaches impose greater financial burden to women due to the multiple visits women require in order to receive screening results and treatment. Assuming a monthly average wage of $360.2 (24), the proportion of monthly income spent on cervical cancer services was estimated by dividing the mean patient cost for each extrapolated screening-and-treatment pathway by the average monthly wage. Based on this approach HPV-LEEP and Pap-LEEP each represented approximately 17% of women’s monthly income, while VIA-LEEP represented approximately 11%. These costs represent a significant share of average monthly income, and may place women at risk of experiencing financial distress when accessing healthcare services.

The average screening turnaround time for HPV DNA testing and Pap-based testing was 32 days, with staff reporting longer delays during key informant interviews. These delays reflect the operational challenges associated with centralized laboratory processing. For both HPV and cytology (Pap smear) testing, samples are processed at the National Reference Laboratory (NRL). These delays are consistent with findings from settings with centralized laboratory processing (30). While HPV DNA testing is often promoted as enabling same-day diagnosis, evidence shows that this is often not feasible. Although few studies in LMIC settings report an HPV turnaround time of 48 h or less, several studies show that HPV testing turnaround times can extend to months (30). Literature also shows that these delays negatively affect health-seeking behavior (15). Reducing turnaround time through same-day or near-point-of-care testing is therefore important for minimizing patient costs.

Across the extrapolated screening and treatment pathways, VIA screening was associated with the lowest patient costs, whereas Pap-LEEP and HPV-LEEP were the most costly. Although absolute cost estimates vary by country, this finding aligns with studies conducted in Kenya, Senegal, and India (9, 11, 31). However, as noted in the literature, VIA is the least costly but has lower sensitivity and specificity, compounded by poor provider interpretation and quality assurance. As a result, while VIA may reduce immediate patient costs, lower diagnostic accuracy may lead to missed precancer cases and ultimately result in downstream preventive inefficiencies and additional costs for patients. These findings underscore the importance of balancing upfront affordability with overall diagnostic performance and long-term efficiency in resource-constrained settings.

Sensitivity analysis revealed that indirect medical costs were the largest cost drivers, followed by productivity costs. These findings align with studies from LMICs such as Kenya and India, where indirect and productivity costs were reported to constitute a significant share of the economic burden of accessing cervical cancer screen-and-treat services (11, 15). Notably, a comparison with the national wage suggested that the extrapolated pathway costs represented a substantial financial burden relative to average monthly income. This finding reflects the financial and economic vulnerability of women seeking cervical cancer screening services. The observed costs must also be interpreted within the broader macroeconomic context, characterized by high unemployment and constrained household incomes. Against the backdrop of a country with a gross domestic product (GDP) per capita of $990.18, an unemployment rate of 30.1%, and 49.7% of the population living below the poverty line, women accessing cervical cancer services incur lost income or forgo essential activities (32). It is therefore important to center screen-and-treat service design around patient needs.

### 4.1 Limitations

The study has several limitations that should be considered when interpreting the results. First, using Lesotho’s latest purchasing power parity (PPP) estimate from 2024 alongside patient cost data collected in 2025 means different years were used, which may influence cost estimates. This uncertainty was not further characterized to generate a range of plausible cost estimates. However, the applied conversion approach is consistent with established practice in health economic evaluations (23).

Second, recall bias was observed among some participants who could not accurately recall arrival times at the health facility or the duration of their visits. As a result, estimates of waiting times and time spent accessing care may be subject to measurement error. Because time-related variables directly influence productivity cost estimates, incorrect recollection may have led to either under- or overestimation of productivity losses. However, recall bias is a common challenge in patient retrospective cost studies, particularly in routine care settings, and the direction of bias is unlikely to systematically alter the relative importance of productivity costs identified in this analysis.

Third, the study population consisted of women who successfully accessed screening and precancer treatment services, which may underestimate the true economic burden faced by the broader target population. Women who did not present for care due to financial, time, or logistical barriers were not captured in this analysis, even though they may have faced even higher relative costs. Consequently, the findings likely represent a lower-bound estimate of patient-incurred costs and may underestimate the deterrent effect of costs on screening uptake and continuity of care.

Finally, the Senkatana Cervical Cancer Center of Excellence serves as both a high-volume referral and walk-in facility, which may influence the patient population accessing services. Women presenting at this center may differ from those attending other health facilities in disease severity, prior health-seeking behavior, socioeconomic characteristics, or geographic proximity. As a result, patient cost estimates from this setting may not be directly generalized to lower-volume facilities or decentralized primary healthcare settings, where patient pathways and cost structures may differ. Despite this limitation, Senkatana is a critical implementation setting for cervical cancer screening in Lesotho, and the findings provide valuable insights into patient costs in a real-world, high-burden service delivery context.

### 4.2 Implications and future directions

This study estimated the patient costs of accessing cervical cancer screen-and-treat strategies at the Senkatana Cervical Cancer Screening Center of Excellence. With the Ministry of Health’s recent adoption of screen-and-treat approaches and primary-based HPV DNA testing, the study addresses a knowledge gap in patients’ resource use. By measuring patient-incurred costs per visit and extrapolating these costs across cervical cancer screening and precancer treatment pathways, this study contributes critical evidence to an underexplored dimension of cervical cancer prevention in LMICs. The findings demonstrate that patient costs remain substantial even within publicly financed service delivery models and may undermine access, continuity of care, and the effectiveness of screening programs. As countries expand HPV DNA testing and screen-and-treat strategies in pursuit of cervical cancer elimination, addressing patient affordability will be essential to translating service availability into population-level impact.

The study’s findings further underscore the importance of accounting for patient costs in the design, implementation, and evaluation of cervical cancer screening programs. Including patient costs as a data point in policy design is critical to ensuring that screen-and-treat strategies are technically and clinically effective and accessible to women in need of services. Although cervical cancer services are provided free of charge at the point of care in Lesotho, women continue to incur substantial indirect costs and productivity losses, highlighting that the removal of user fees alone is insufficient to ensure equitable access to screening and treatment services. As Lesotho continues to scale up HPV DNA testing as part of the WHO cervical cancer elimination strategy, greater attention should be given to strengthening laboratory systems, reducing turnaround times for screening results, and expanding access to same-day diagnosis and treatment where feasible. Addressing health system constraints, including laboratory capacity, diagnostic platform placement, and human resource shortages, will be essential to reducing patient costs and ensuring that cervical cancer screening programs are not only clinically effective but also financially accessible to the women they are intended to serve.

## Data Availability

The raw data supporting the conclusions of this article will be made available by the authors, without undue reservation.
