# Supplementary material for: A patient costing analysis of Lesotho’s cervical cancer screening program
Source: Front Public Health. 2026 Oct 14;14:1888325. doi: 10.3389/fpubh.2026.1888325 (PMC13713295; doi:10.3389/fpubh.2026.1888325)
Supplement: Supplementary file 1 [file Supplementary_file_1.docx]

| **#** | **Question** | **Response** |
| --- | --- | --- |
| **Sociodemographic Characteristics** | | |
| 1 | How old are you? | Input age |
| 2 | What is your marital status? | a.Single  b.Co-habiting  c.Married  d.Divorced  e. Other |
| 3 | What is your employment status? | a.Unemployed  b.Part-time employed  c.Full-time employed  d. Self-employed |
| 4 | What is your occupation? | *Input occupation* |
| 5 | What is your education status? | a.None  b.Primary education  c.Secondary education  d.Tertiary education |
| 6 | What is the family size of your immediate family? | Input # of members |
| Health-seeking behavior | | |
| 7 | Is this your first-time receiving services at the cervical cancer centre? | a.Yes  b.No |
| 8 | Which service did you receive at the center today? | a. Screening b. LEEP |
| 9 | If you were screened, did you receive your screening results on the same day? | a. Yes b. No |
| 10 | If no to the question above, when will you collect the results? | *Input date* |
| 11 | Will you travel to the center again to collect the screening results? | a. Yes b. No |
| Travel to Sankatana Cervical Cancer Centre (Indirect Costs) | | |
| 12 | How did you get to Sankatana Cervical Cancer Centre today? | a. I walked to the center  b.I used public transport.  c. I used private transport |
| 13 | How long did it take to travel to the centre? | *Input time* |
| 14 | What time did you arrive at the center? | *Input time* |
| 15 | What time would you have wanted to leave? | *Input time* |
| Other Indirect Costs | | |
| 16 | Did you come to Sankatana alone, or did someone accompany you? | a . I came alone  b. someone accompanied me |
| 17 | Did you have to spend money to come to the clinic for screening today? | a. Yes  b. No |
| 18 | If yes, what did you spend money on? |  |
| 19 | How much did you spend? | *input amount* |
| 20 | How much did you pay to travel to the center? | *Input amount* |
| 21 | If not, did someone else have to spend any money for you to come to the clinic for screening today? | a.Yes  b.No |
| 22 | If yes, what did they spend money on? | *Input amount* |
| 23 | How much did they spend? | *Input amount* |
| 24 | How long did you wait at the center before receiving services? | Number of hours |
| 25 | Did you have to alter your typical plans to come to the clinic for screening? | a.Yes  b.No |
| 26 | If yes, in what way (s)? |  |
| 27 | Has this meant a loss of income for you? | a. Yes  b. No |
| 28 | If yes, how much? | *Input amount* |
| 29 | Has this meant a loss of income for anyone else? | a.Yes  b.No |
| 30 | If yes, how much? | *Input amount* |
